# Corrigendum: Origin and structure of polar domains in doped molecular crystals

**DOI:** 10.1038/ncomms14597

**Published:** 2017-03-06

**Authors:** E. Meirzadeh, I. Azuri, Y. Qi, D. Ehre, A. M. Rappe, M. Lahav, L. Kronik, I. Lubomirsky

Nature Communications 7: Article number: 13351; DOI: 10.1038/ncomms13351 (2016); Published 11 08 2016; Updated 03 06 2017

In Table 2 of this Article, the graph displaying ‘Pyroelectric coefficient versus temperature' for 0.03% L-phenylalanine incorrectly replicates the graph above. The correct version of Table 2 appears below as Table 1.

## Figures and Tables

**Table 1:**
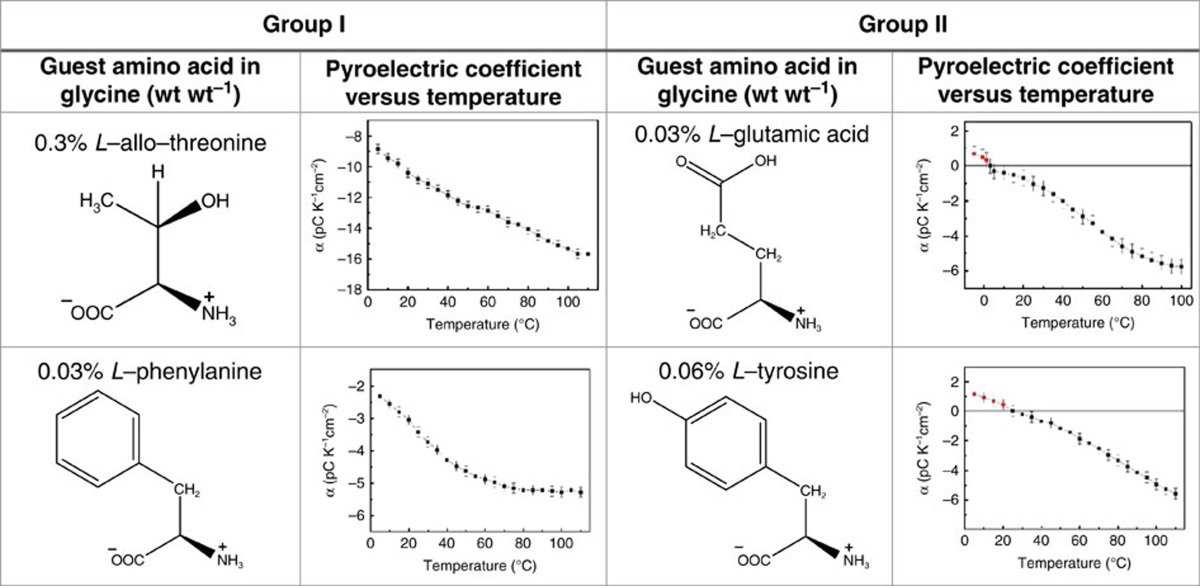
Temperature dependence of the pyroelectric coefficient.

Group I: Pyroelectric coefficient does not change sign with temperature; Group II: Pyroelectric coefficient changes sign from positive to negative upon heating. The error bars show the s.d. of the pyroelectric coefficient at each temperature. Error bars represent s.e.m. values.

