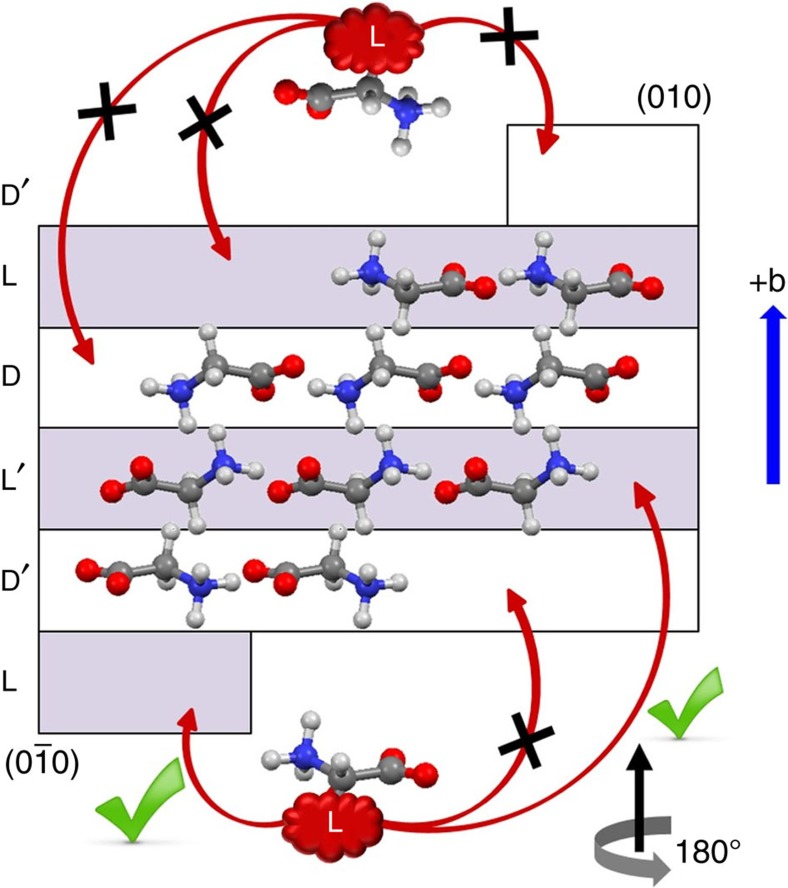# Erratum: Origin and structure of polar domains in doped molecular crystals

**DOI:** 10.1038/ncomms15590

**Published:** 2017-05-09

**Authors:** E. Meirzadeh, I. Azuri, Y. Qi, D. Ehre, A. M. Rappe, M. Lahav, L. Kronik, I. Lubomirsky

Nature Communications 7: Article number: 13351; DOI: 10.1038/ncomms13351 (2016); Published: 11 08 2016; Updated: 05 09 2017

In Fig. 1 of this Article, the top crystallographic face of the crystal was inadvertently mislabelled ‘(01̄0)’ during the production process. It should read ‘(010)’. The correct version of Fig. 1 appears below.

## Figures and Tables

**Figure 1:**