# Supplementary material for: Origin and structure of polar domains in doped molecular crystals
Source: Nat Commun. 2016 Nov 8;7:13351. doi: 10.1038/ncomms13351 (PMC5105173; doi:10.1038/ncomms13351)
Supplement: Supplementary Information — Supplementary Figures 1-7, Supplementary Tables 1-2, Supplementary Discussion and Supplementary References. [file ncomms13351-s1.pdf]

Supplementary Figures

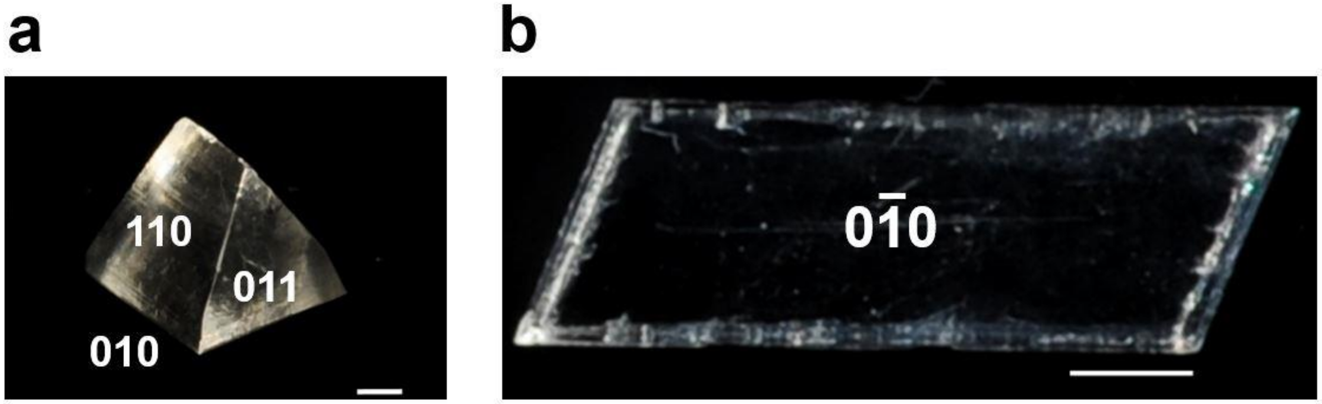

Supplementary Figure 1 | Photographs of the amino acid crystals. (**a**) Pure crystal of α-glycine where the $(0\bar{1}0)$ face develops at the solution/glass interface (represents the base of the pyramid). (**b**) α-glycine doped with ~0.3% of *L*-amino acid. Scale bars, 1 mm.

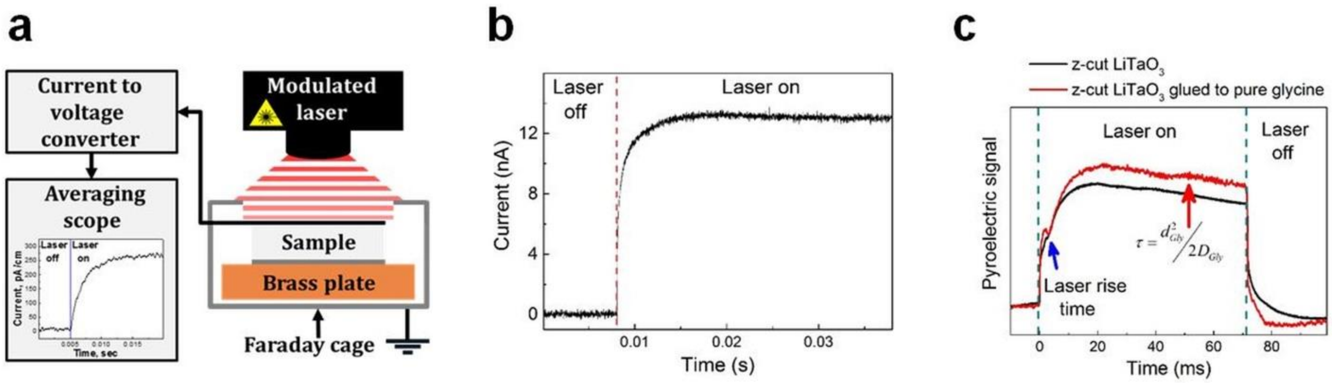

Supplementary Figure 2 | Pyroelectric measurement. (**a**) Schematic illustration of the periodic temperature change technique. (**b**) Pyroelectric signal from a homogenous polar crystal. (**c**) Pyroelectric current of $LiTaO_3$ glued with pure α-glycine crystal.

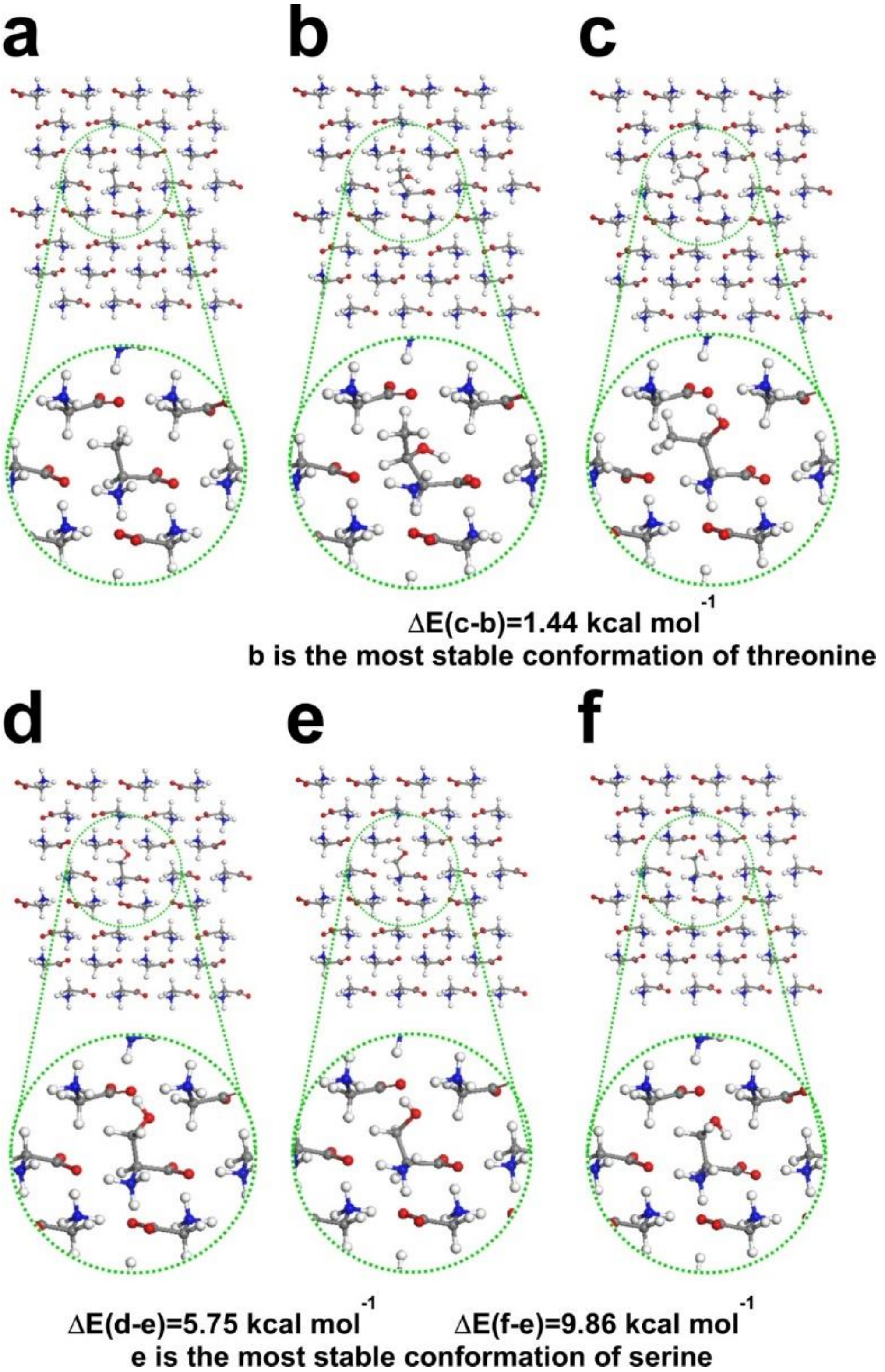

Supplementary Figure 3 | Metastable conformations of the guest and the arrangement of the host glycine molecules in the vicinity of the guest site. (**a**) α-Glycine doped with 0.78% *L*-alanine (Supplementary Software File 1), (**b**) and (**c**) with *L*-threonine (Supplementary Software Files 2 and 3), (**d**), (**e**) and (**f**) with *L*-serine (Supplementary Software Files 4, 5 and 6).

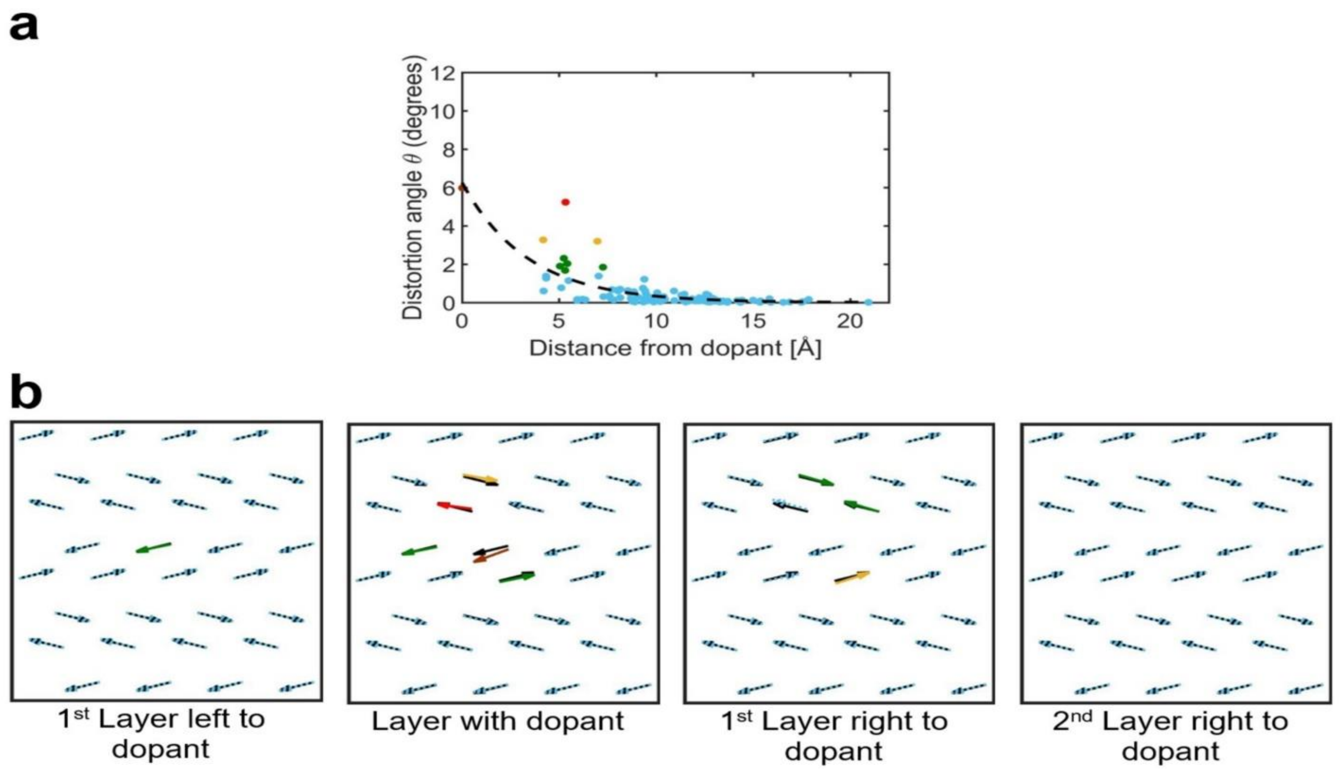

Supplementary Figure 4 | Distortion of the molecules in the super cell, induced by 0.78% *L*-alanine to the α-glycine crystal. (**a**) Distortion angle as function of the distance from the α-carbon of the dopant to the α-carbon of the host molecules. The colors represent the degree of distortion angle due to the presence of the dopant. The dopant is colored by brown dot. (**b**) 2D Deflection graphs of the C(carboxylic)-N vector relative to the undoped crystal. The brown arrow represents the dopant molecule.

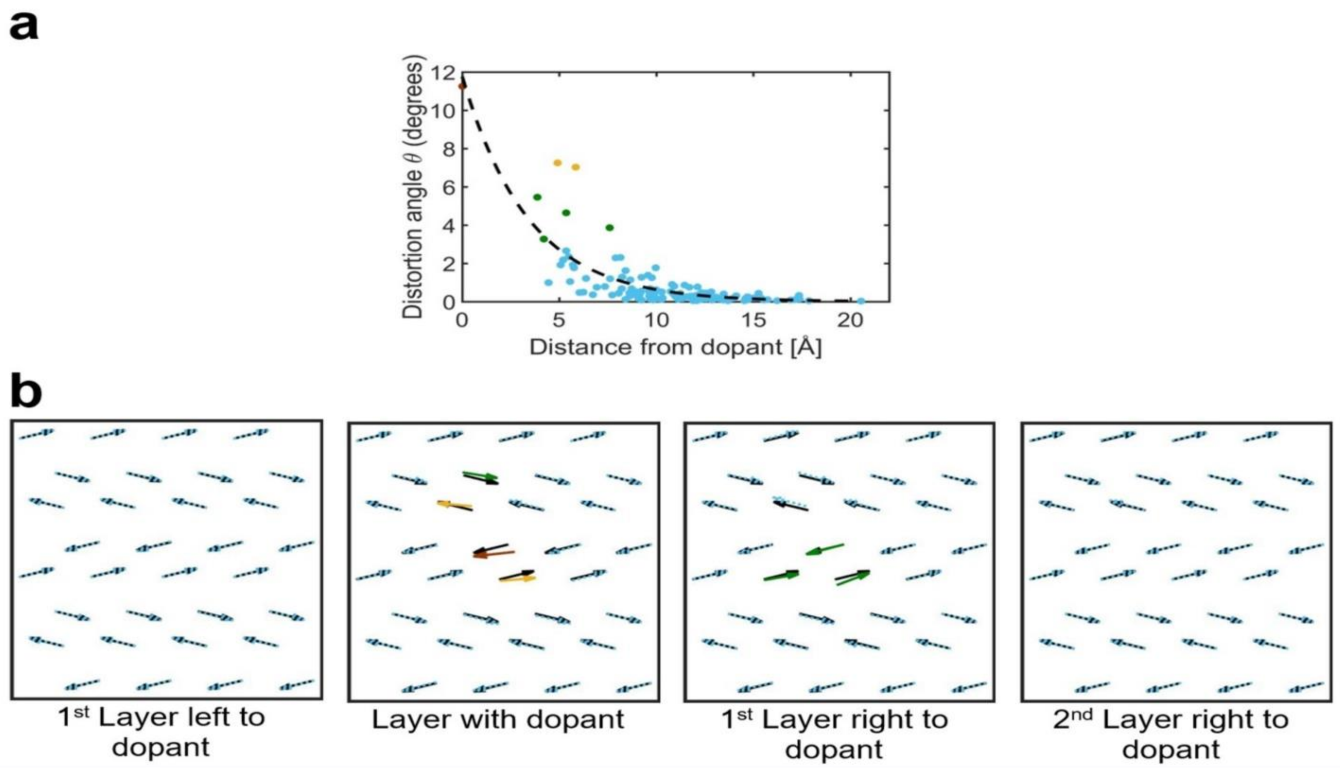

Supplementary Figure 5 | Distortion of the molecules in the super cell, induced by 0.78% *L*-threonine to the α-glycine crystal. (**a**) Distortion angle as function of the distance from the α-carbon of the dopant to the α-carbon of the host molecules. The colors represent the degree of distortion angle due to the presence of the dopant. The dopant is colored by brown dot. (**b**) 2D Deflection graphs of the C(carboxylic)-N vector relative to the undoped crystal. The brown arrow represents the dopant molecule.

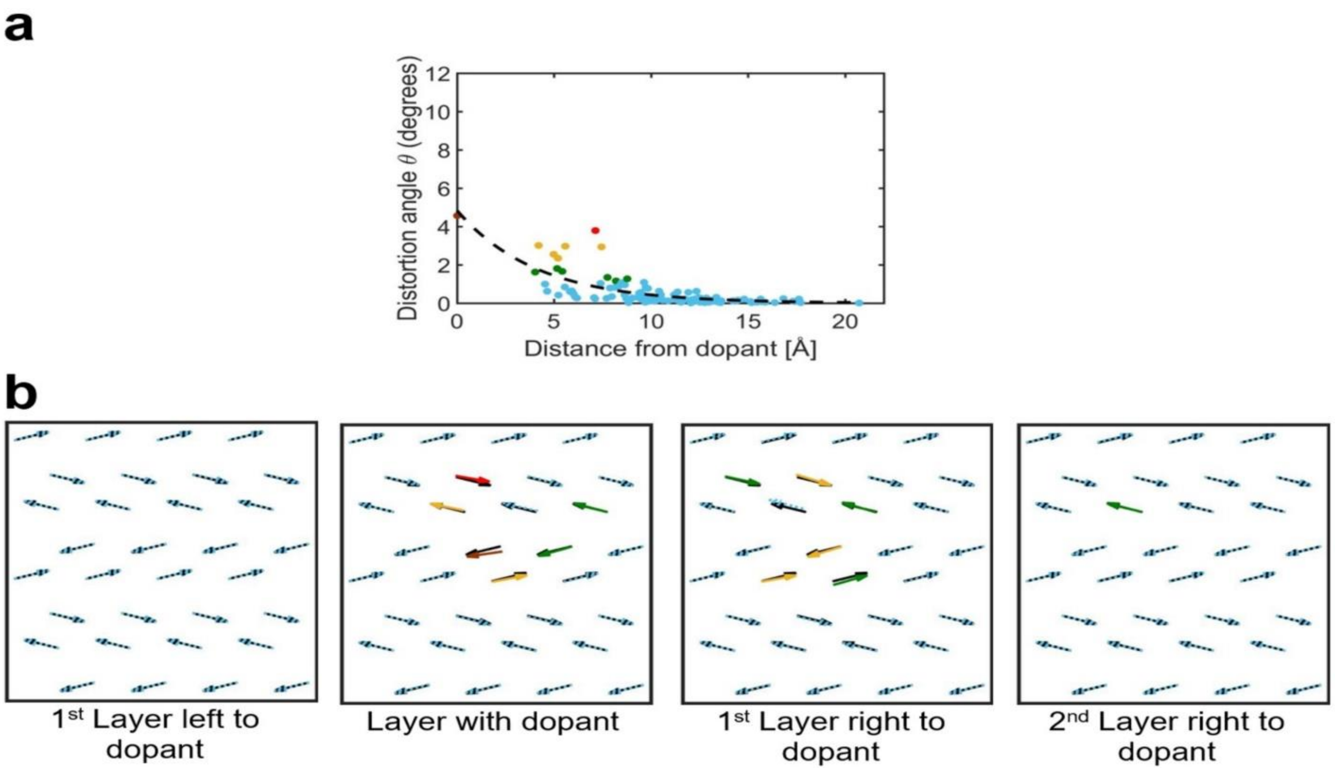

Supplementary Figure 6 | Distortion of the molecules in the super cell, induced by 0.78% *L*-serine to the α-glycine crystal. (**a**) Distortion angle as function of the distance from the α-carbon of the dopant to the α-carbon of the host molecules. The colors represent the degree of distortion angle due to the presence of the dopant. The dopant is colored by brown dot. (**b**) 2D Deflection graphs of the C(carboxylic)-N vector relative to the undoped crystal. The brown arrow represents the dopant molecule.

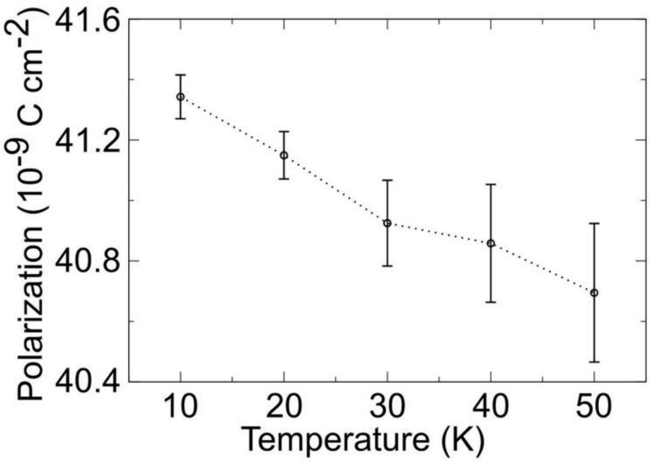

Supplementary Figure 7 | MD-computed temperature-dependent polarization for the configuration of serine with an intra-molecular H-bond. The dots are average values and the error bars represent the SD.

Supplementary Tables

Supplementary Table 1 | Pyroelectric coefficient at 25 °C as function of dopant concentration.

Face Measured	Alanine Doped Glycine		Threonine Doped Glycine		Serine Doped Glycine	
	Alanine Conc. [wt wt^{-1}]	Pyroelectric Coefficient [C $K^{-1}cm^{-2}$]	Threonine Conc. [wt wt^{-1}]	Pyroelectric Coefficient [C $K^{-1}cm^{-2}$]	Serine Conc. [wt wt^{-1}]	Pyroelectric Coefficient [C $K^{-1}cm^{-2}$]
**Top**	0.32%	-10.9×10^{-12}	0.32%	-12.6×10^{-12}	0.32%	11.0×10^{-12}
**Cleaved #1**	0.11%	-4.5×10^{-12}	0.12%	-4.6×10^{-12}	0.14%	2.9×10^{-12}
**Cleaved #2**	0.09%	-3.8×10^{-12}	0.10%	-3.7×10^{-12}	0.10%	2.1×10^{-12}

The top surface of the crystal is enriched with dopant, while upon cleaving a more homogeneous mixed crystal is revealed.

Supplementary Table 2 | Polarization contributions along the *b*-axis from MD simulations at 0 K.

Unit (10^{-9} C cm^{-2})	Guest Polarization Contribution	Host Polarization Contribution
Alanine	29.80	-9.72
Threonine	46.47	31.98
Serine	-23.36	84.44

Supplementary Discussion

Crystal growth. A supersaturated solution (130%) of glycine with the dopant was prepared by dissolving the amino acids in water (Ultra-pure Millipore water, 18.2 MΩ cm at 25 °C, Millipore Synergy UV, Type 1 water) and heating it to ~80 °C, allowing for full dissolution. The solutions were filtered through cotton wool into glass crystallization growth dishes which were covered with filter paper for allowing slow evaporation. Large transparent single crystals were chosen, washed in water, and dried. The crystalline structure was verified and major faces were indexed using an Ultima-III (sealed X-ray tube, Cu anode, 3 kW, RIGAKU, Japan) X-ray diffractometer. The {010} faces in the pure glycine crystal developed at the glass-water interface since no mass transfer of solute molecules was possible to the surface of the growing crystal that was directly in contact with the glass. The doped crystals exhibit plate-like morphology with 0.5-1 mm thickness and a 0.05-0.2 cm^2 area of the top surface $(0\bar{1}0)$ (Supplementary Figure 1). All crystals were heated to 100 °C for 2 hours in order to remove the surface pyroelectric signal, originating from the wetted surfaces of the crystals[1].

HPLC determination of the dopant concentration. The pure glycine crystal was free from other amino acids. The concentration of the dopant as function of the depth was determined by pyroelectric and HPLC (high pressure liquid chromatography) measurements on crystalline segments, cleaved perpendicular to the polar *b*-axis. The cleaved segments were diluted in 1 ml of water (Ultra-pure Millipore water, 18.2 MΩ cm at 25 °C, Millipore Synergy UV, Type 1 water), filtered, diluted × 10, and subjected to amino acid analysis. The analysis was performed on a Waters Alliance 2695 Instrument, equipped with a Waters Milford Mass 474 fluorescence detector, using the Waters Milford Mass AccQ-Tag amino acids analysis kit. The column was AccQ-Tag, reversed phase, 150×3.9 mm. The samples were reacted for fluorescence detection with an AccQ-Tag Analysis kit. The concentration of the guest molecules was within the range 0.03-0.3% wt wt^{-1}. For (*L*-) alanine, threonine, allo-threonine and serine, the dopant concentration was ≈0.3% wt wt^{-1}, and for phenylalanine, tyrosine and glutamic acid 0.03-0.06% wt wt^{-1}. The HPLC analysis of the cleaved-off parts confirmed that the dopant content decreases with depth. The pyroelectric current measured after each cleave was correspondingly lower, proportionally to the decrease in the dopant concentration, for α-glycine doped with alanine, threonine and serine (Supplementary Table 1). In the case of tyrosine, phenylalanine, and glutamic acid, the dopant was detected only in the segments residing within ~100-200 µm from the top surface.

Pyroelectric measurement. The pyroelectric current of the mixed crystal was measured by the periodic temperature change technique[2] (Chynoweth method (Supplementary Figure 2a)). Once the sample starts losing heat to its substrate, the current starts to decay. Therefore, if the heating time is shorter than the thermal diffusion time through a homogeneous crystal, the current will remain constant as a function of time (Supplementary Figure 2b). For further information, see Appendix 2e in ref. 2. The top contact was completely opaque, providing heat entrance exclusively by thermal diffusion through the surface.

Estimation of the heat diffusion coefficient of α-glycine. The heat diffusion coefficient, *D*, can be calculated from the pyroelectric current, *J*[3]. Here we estimate this value using similar logic. The current is proportional to the temperature change with time[2]: $J = \alpha \cdot \partial T/\partial t$, where α is the pyroelectric coefficient. The derivative of the temperature with respect to time is[4]:

$$\text{(S1)} \quad \frac{\partial T}{\partial t} = \frac{F_d}{C\sqrt{\pi D t}} \cdot \exp\left(-\frac{x^2}{4Dt}\right),$$

where F_d is the heat flux at the surface and C is the thermal capacitance per unit volume. In order to calculate the heat diffusion coefficient of α-glycine crystal, we measured the pyroelectric current of a well-known polar material (z-cut $LiTaO_3$) after gluing a pure α-glycine crystal (which is not pyroelectric) to the top surface of the $LiTaO_3$ crystal. The current should reach to its maximum value when $t \equiv \tau = {d^2_{\mathrm{Gly}}}/{2D_{\mathrm{Gly}}}$ (Supplementary Figure 2c) where d=0.022 cm is the thickness of the α-glycine crystal. The heat diffusion coefficient of α-glycine according to this measurement is $D \sim 0.05$ cm^2/s.

Modelling using Dispersion-Corrected DFT. We provided different initial geometries for the guest molecules (*L*-alanine, *L*-serine, and *L*-threonine) in the ideal host crystal, and relaxed the overall structure. The calculations showed one locally stable conformation for alanine, two for threonine and three for serine (see Supplementary Figure 3 and Supplementary Software Files 1-6 for details). The angular distortion that the dopant induces to its neighboring glycine molecules is represented in Supplementary Figure 4, 5 and 6.

We used the lowest-energy conformation of each host-guest structure for calculating the polarization and the pyroelectric coefficient. The polarization at different temperatures was calculated by the Berry Phase method[5]. In the modern theory of polarization[5], it is recognized that the polarization of a periodic solid cannot be extracted from the charge density in the density in the unit cell but is instead a property of the wave functions. In a periodic cell, the position operator **r** is not well-defined and so the polarization is calculated as the expectation value of d/dk; this makes the polarization a Berry's phase in momentum space. In this study we focus on polarization along the b axis, P_b. The polarization component along a lattice vector is defined modulo a polarization quantum, $P_Q=e/S$, where e is the electron charge and S is the surface area of the unit cell bounded by $\boldsymbol{a}$ and $\boldsymbol{c}$, $S=\boldsymbol{a}\times\boldsymbol{c}\cdot\boldsymbol{b}/b$, where $b=|\boldsymbol{b}|$[5, 6]. When calculating quantities involving polarization differences, the polarization quanta must be handled with care. For systems involving continuous change, the polarization should change continuously as well, enabling unambiguous calculation of differences.

Here, when polarization is calculated with different lattice vector lengths and temperature-dependent $S(T)$, the polarization quantum itself, $P_Q(T)$, changes and the customary cancellation of quanta must be generalized. For calculated $P(T)$ the polarization can be $P(T)+nP_Q(T)$, where n is the polarization quantum. In the experiment, the sample rests for a long time at a particular temperature T_0, long enough for it to gather a compensating charge per unit cell of $Q=-[P(T_0)+nP_Q(T_0)]S(T_0)=-P(T_0)S(T_0)-ne$ on its boundaries. Thus, at T_0 the total (bound polarization plus compensating) charge is zero. Then, the temperature is changed, changing the polarization and the axis lengths. At temperature T, the total surface charge per cell is $[P(T)+nP_Q(T)]S(T)+Q=P(T)S(T)-P(T_0)S(T_0)$. This makes the total polarization $P(T)-P(T_0)S(T_0)/S(T)$, including bound and compensating charges. Differentiating this expression yields the pyroelectric coefficient:

$$\text{(S2)} \quad \alpha = \frac{dP(T)}{dT} + \frac{P(T_0)S(T_0)}{S^2(T)} \cdot \frac{dS(T)}{dT}$$

This expression includes the change in the bulk polarization as lattice parameters are changed owing to temperature, as well as account for the change in compensating charge density brought about solely by deforming the unit cell.

Supplementary References

1. Piperno, S. et al. Water-Induced Pyroelectricity from Nonpolar Crystals of Amino Acids. *Angewandte Chemie-International Edition* **52**, 6513-6516 (2013).
2. Lubomirsky, I. & Stafsudd, O. Practical guide for pyroelectric measurements. *Rev. Sci. Instrum.* **83**, 051101 (2012).
3. Muensit, S. & Lang, S.B. Pyroelectric technique for measurement of thermal diffusivity of thin solid materials. *Ferroelectrics* **293**, 341-350 (2003).
4. Ehre, D., Mirzadeh, E., Stafsudd, O. & Lubomirsky, I. Pyroelectric Measurement of Surface Layer: The Case of Thin Film on Dielectric Substrate. *Ferroelectrics* **472**, 41-49 (2014).
5. Resta, R. & Vanderbilt, D. Theory of polarization: A modern approach. *Physics of Ferroelectrics: A Modern Perspective* **105**, 31-68 (2007).
6. Djani, H., Bousquet, E., Kellou, A. & Ghosez, P. First-principles study of the ferroelectric Aurivillius phase Bi2WO6. *Phys. Rev. B* **86**, 054107 (2012).
